# Trapped Ion Oscillation Frequencies as Sensors for Spectroscopy

**DOI:** 10.3390/s100302169

**Published:** 2010-03-16

**Authors:** Manuel Vogel, Wolfgang Quint, Wilfried Nörtershäuser

**Affiliations:** 1 Department of Physics, Imperial College London, SW7 2AZ, London, UK; 2 GSI Helmholtzzentrum für Schwerionenforschung, D-64291 Darmstadt, Germany; E-Mail: w.quint@gsi.de; 3 Nuclear Chemistry Department, Johannes-Gutenberg-Universität, D-55099 Mainz, Germany; E-Mail: w.noertershaeuser@uni-mainz.de

**Keywords:** penning trap, magnetic bottle, spectroscopy, magnetic moment

## Abstract

The oscillation frequencies of charged particles in a Penning trap can serve as sensors for spectroscopy when additional field components are introduced to the magnetic and electric fields used for confinement. The presence of so-called “magnetic bottles” and specific electric anharmonicities creates calculable energy-dependences of the oscillation frequencies in the radiofrequency domain which may be used to detect the absorption or emission of photons both in the microwave and optical frequency domains. The precise electronic measurement of these oscillation frequencies therefore represents an optical sensor for spectroscopy. We discuss possible applications for precision laser and microwave spectroscopy and their role in the determination of magnetic moments and excited state life-times. Also, the trap-assisted measurement of radiative nuclear de-excitations in the X-ray domain is discussed. This way, the different applications range over more than 12 orders of magnitude in the detectable photon energies, from below *μ*eV in the microwave domain to beyond MeV in the X-ray domain.

## 1. Introduction

Penning traps usually serve as a mere means for ion confinement under well-defined conditions. For spectroscopic applications, localization of ions and the possibility to cool their motion to very low velocities are the main features. However, as will become evident later, also specific properties of the ion confinement and the interaction between the ions and the storing fields can be employed for advanced spectroscopy.

Spectroscopy in charged particle traps such as Penning traps has widely been used for precision spectroscopy of stored ions at low velocities, as has been detailed out in [1,2]. Laser spectroscopy of atomic or molecular transitions is most often performed by detection of fluorescence photons upon resonant laser excitation [2]. This is also true for confined particles in traps, which have been used for precise determinations of atomic transition frequencies [3–5] and of fundamental quantities [6–10].

Laser and microwave sources for excitation of transitions are available within a broad range of frequencies [2] and feature bandwidths down to the sub-Hz domain [11,12], whereas the detection of fluorescence photons emitted by trapped particles may be very difficult or even impossible due to a lack of well-suited detectors which can be operated in strong magnetic fields and at cryogenic (liquid helium) temperatures. Furthermore, especially in the infrared domain, photon detectors often suffer from small quantum efficiencies, small sensitive areas and high dark count rates.

It is therefore desirable to have alternative detection schemes at hand which are non-optical. Confined ions in a Penning trap, typically single ions, offer such a possibility. When the confining fields are chosen in such a way that the ion oscillation frequencies in the trap depend unambiguously on the energy of the ion motion, they may serve as an electronic detector for the absorption or emission of microwave, optical, or X-ray photons, as will be detailed out below.

The well-known continuous Stern-Gerlach effect [13,14] is an example of such an application. The ion oscillation frequency serves as a measure for the spin direction of an electron bound in the ion [15]. This way, an electronic radiofrequency measurement of a macroscopic ion oscillation serves as a sensor for microwave photon absorption by the ion and the subsequent spin flip of the bound electron. In the following, we will discuss this and other examples both from atomic and nuclear physics and provide a systematic account of the underlying trap physics.

## 2. Ion Oscillation in a Penning Trap

### 2.1. Ion Motion in Ideal Fields

The confinement of ions in a Penning trap [1] is assured by the Lorentz force in a homogeneous magnetic field forcing the particles on a cyclotron trajectory around the field axis. Thus, the ions are confined in the two dimensions perpendicular to the magnetic field axis. Confinement in the axial dimension is achieved by a superimposed quadrupolar electrostatic potential along the magnetic field axis.

**Figure 1.**
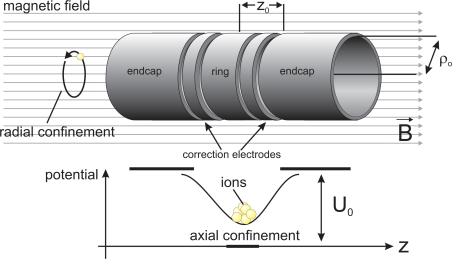
Typical geometry of a cylindrical Penning trap with open endcaps and correction electrodes between the central ring and the endcap electrodes. The confinement of ions by electric and magnetic fields is indicated schematically. The trapping region is located around the centre of the arrangement, inside the hollow cylinder electrodes. Details are given in [16].

Figure 1 schematically shows a typical cylindrical Penning trap with open endcaps and a pair of correction electrodes used to maximize the harmonicity of the electrostatic trapping potential. The properties of such a trap are described in detail in [16]. Each confined ion moves on a three-dimensional trajectory composed of three individual motions, namely the “modified” cyclotron motion, the axial oscillation in the electrostatic potential well and a drift motion in the crossed electric and magnetic fields named “magnetron motion” [17].

In a so-called “ideal” Penning trap, *i.e.*, in the absence of inhomogeneities and anharmonicities, the axial oscillation frequency of a confined ion is given by

(1) $$\omega_{z}=\sqrt{\frac{qU_{0}C_{2}}{{\mathrm{md}}^{2}}}\;\;\;\;\;\;\text{with}\;\;\;\;\;\;d^{2}=\frac{1}{2}\left(z_{0}^{2}+\frac{1}{2}\rho_{0}^{2}\right)$$

where *q* is the ion charge, *U*_0_ is the electric trapping potential, *m* is the ion mass and *d* is the characteristic trap dimension defined by the inner trap radius *ρ*_0_ and the axial trap size *z*_0_. The factor *C*_2_ equals unity in the ideal Penning trap, imperfections will be explained below. The two superimposed radial oscillation frequencies are given by

(2) $$\omega_{+}=\frac{\omega_{c}}{2}+\sqrt{{\left(\frac{\omega_{c}}{2}\right)}^{2}-\frac{\omega_{z}^{2}}{2}}$$

(3) $$\omega_{-}=\frac{\omega_{c}}{2}-\sqrt{{\left(\frac{\omega_{c}}{2}\right)}^{2}-\frac{\omega_{z}^{2}}{2}}$$

where *ω*_+_ is called the “modified cyclotron frequency” and *ω*_−_ is the “magnetron drift frequency”. *ω_c_* is the frequency of the ion motion perpendicular to the magnetic field *B* in absence of electric forces, the so-called “unperturbed cyclotron frequency” given by $\omega_{c}=\frac{q}{m}\;B$. The three characteristic frequencies are related by an invariance theorem stating that $\omega_{+}^{2}+\omega_{-}^{2}+\omega_{z}^{2}=\omega_{c}^{2}$ [18,19]. However, the energies and therefore the amplitudes of the oscillations are unrelated and energy transfer between motional modes is negligible. Hence, a specific motion can be cooled or heated without affecting the remaining motions. Cooling means a reduction of the motional temperature of the ion(s). The assignment of a temperature even to a single confined particle’s motion is possible in a straightforward way and has been discussed for example in [20]. As far as cooling of the axial and cyclotron motions is concerned, the expressions energy, temperature, oscillation amplitude and mean velocity can be used synonymously. In an ideal Penning trap, *i.e.*, in a perfectly homogeneous magnetic field and a purely quadrupolar electrostatic potential, the three characteristic frequencies are determined only by the confining fields and intrinsic ion properties like mass and charge, and depend on the motional energies only through the relativistic effect. This changes with the introduction of additional field components.

### 2.2. Coupling of Oscillations through Field Imperfections

The presence of either a magnetic or electrostatic imperfection represents a coupling of the oscillatory motions such that the individual oscillation frequencies become dependent on the energies of all motions. This is due to the fact that in imperfect fields the effective field strength (which determines the oscillation frequencies) experienced by the ion depends on its position in the trap and deviates from the ideal value when the energy (amplitude) of a motion changes. Using the hierarchy *ω*_−_ ≪ *ω_z_* ≪ *ω*_+_ of the frequencies [16], this dependence can be expressed in a classical formalism. We will follow the discussion in [17] and use the matrix equation

(4) $$\left(\begin{array}{c} \Delta \omega_{+}/\omega_{+}\\ \Delta \omega_{z}/\omega_{z}\\ \Delta \omega_{-}/\omega_{-}\\ \Delta \omega_{L}/\omega_{L} \end{array}\right)=(M_{E}+M_{B}+M_{R})\;\left(\begin{array}{c} E_{+}\\ E_{z}\\ E_{-} \end{array}\right)$$

where *M_E_* and *M_B_* are 3 × 4 matrices containing the electric and magnetic dependences, respectively. The matrix *M_R_* contains the relativistic corrections. Here, *ω_L_* is the Larmor frequency of the particle, *i.e.*, the frequency of spin precession around the magnetic field axis. Since it is an intrinsic ion property, it is not an oscillatory motion in the same sense as the above trapping motions, however, it shows dependences which can be described within the same formalism.

#### 

##### Electrostatic Anharmonicity

Let the anharmonicity of the electrostatic potential near the trap centre be described by the expansion [16]

(5) $$U=\frac{1}{2}U_{0}\sum_{k=0}^{\infty }\;C_{k}\;{\left(\frac{r}{d}\right)}^{k}\;P_{k}(\cos\theta )$$

where the *P_k_*(*cosθ*) are Legendre polynomials and *r* is the ion distance from the electric trap centre. For this discussion, it is sufficient to include the dimensionless expansion coefficient *C*_2_ and to account for electric imperfections characterized by *C*_4_. Higher-order contributions are considered as negligible. The next term *C*_6_ is suppressed with respect to the term in *C*_4_ by a factor of (*r*/*d*)^2^, which typically is of order 10^−4^ or smaller. Odd terms vanish because of the point symmetry with respect to the trap centre. The coefficients including *C*_4_ depend on applied voltages and can be written as [16]

(6) $$C_{2}=C_{2}^{\left(0\right)}+D_{2}\frac{U_{C}}{U_{0}}$$

(7) $$C_{4}=C_{4}^{\left(0\right)}+D_{4}\frac{U_{C}}{U_{0}}$$

where the $C_{k}^{\left(0\right)}$ and *D_k_* are given by the trap geometry [16] and *U_C_* is the voltage applied to the correction electrodes of the trap. Whereas *C*_2_ only represents a linear scaling of the trapping potential, a non-vanishing term *C*_4_ ≠ 0 leads to frequency dependences described by

(8) $$M_{E}=\frac{6C_{4}}{{\mathrm{qU}}_{0}}\left(\begin{array}{ccc} \eta^{4}/4 & -\eta^{2}/2 & -\eta^{2}\\ -\eta^{2}/2 & 1/4 & 1\\ -\eta^{2} & 1 & 1\\ 0 & 0 & 0 \end{array}\right).$$

Here, *η* = *ω_z_/ω*_+_. The bottom line of the matrix *M_E_* contains only zeros since the Larmor frequency is a purely magnetic property and thus not affected by electric anharmonicities.

##### Magnetostatic inhomogeneity: magnetic bottle

Let the symmetry axis *ê_z_* of the trap be parallel to the magnetic field *B*_0_ and the radial coordinate be *ρ* (see also Figure 1). The magnetic field near the centre of a trap can then be written as

(9) $$B(z,\;\rho )=B_{0}-2B_{1}z+B_{2}\;\left(z^{2}-\frac{1}{2}\rho^{2}\right)+\ldots$$

where *B*_0_ is the homogeneous part of the field, *B*_1_ describes a *z*-dependence (linear gradient) and *B*_2_ characterizes a so-called “magnetic bottle” and its dependence on both an axial and a radial coordinate. Higher-order terms are not of relevance for the present discussion. A magnetic bottle is therefore a magnetic field inhomogeneity of the kind ${\vec{B}}^{\prime }=B_{2}((z_{0}^{2}-\rho_{0}^{2}/2){\hat{e}}_{z}-z{\vec{\rho }}_{0})$ with *B*_2_ ≠ 0 superimposed on the magnetic trapping field *B*_0_ with radial symmetry around the trap centre. The presence of *B*_2_ ≠ 0 results in a dependence of the oscillation frequencies on the motional energies given by

(10) $$M_{B}=\frac{1}{{2m\omega }_{-}\omega_{+}}\;\frac{B_{2}}{B_{0}}\;\left(\begin{array}{ccc} -\eta^{2} & 1 & 2\\ 1 & 0 & -1\\ 2 & -1 & -2\\ -\eta^{2} & 1 & 2 \end{array}\right).$$

This means that all frequencies depend linearly on all energies except for the axial frequency which does not depend on the axial energy (the corresponding matrix element is zero).

##### Relativistic Shifts

Relativistic frequency shifts can be understood in terms of the relativistic mass shift with the kinetic energy in the respective motion and lead to

(11) $$M_{R}=-\frac{1}{{\mathrm{mc}}^{2}}\left(\begin{array}{ccc} 1 & 1/2 & -\eta^{2}\\ 1/2 & 3/8 & -\eta^{2}/4\\ -\eta^{2} & -\eta^{2}/4 & -\eta^{4}/4\\ 2/9 & 1/2 & -\eta^{2} \end{array}\right).$$

**Figure 2.**
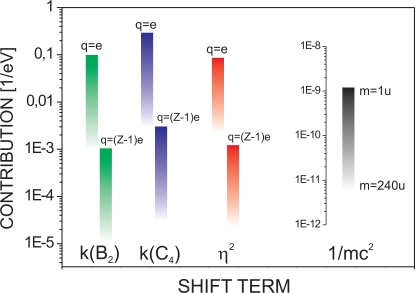
Typical values of *k*(*C*_4_), *k*(*B*_2_) and *η*^2^ both for singly charged (*q* = *e*) and hydrogen-like ions with a charge of *q* = (*Z* − 1)*e*. The relativistic term 1/(*mc*^2^) is shown for masses between *m*=1 u and *m* = 240 u. For details see text.

For the three ion oscillations, this is a straightforward transformation acting on the motional frequencies through the mass shift. Following the discussion in [22–24], for the relativistic effect on the Larmor precession frequency, in principle two cases need to be distinguished, depending on the spin orientation relative to the magnetic field. Writing *β* = *v*/*c* with the ion velocity *v* and *γ* = (1 − *β*^2^)^−1/2^, the free ion cyclotron frequency is given by *ω_c_* = *qB*/(*γm*). Denoting the bound electron’s magnetic moment anomaly by *a* = *g*/2 − 1 one finds the Larmor frequency to be $\omega_{L}=(1+a)\omega_{c}^{e}$ for an orientation parallel to the magnetic field, whereas perpendicularly one finds $\omega_{L}=(1+\gamma a)\omega_{c}^{e}$. However, since for ions at about 4 K the factor *γ* is of order 10^−15^, this effect will be neglected. Typical values for both the shift terms *k*(*C*_4_) := 6*C*_4_/(*qU*_0_) and *k*(*B*_2_) := *B*_2_/(2*mω*_−_*ω*_+_ *B*_0_) in equations (8) and (10) range from 10^−5^/eV to 10^−3^/eV in highly charged ions and from 10^−3^/eV to 10^−1^/eV in singly charged ions. The value of *η*^2^ can be approximated by ${\mathrm{mU}}_{0}/({\mathrm{qd}}^{2}\;B_{0}^{2})$ and typically ranges from 10^−5^ to 10^−3^ in highly charged ions and from 10^−3^ to 10^−1^ in singly charged ions. The relativistic term 1/(*mc*^2^) is of order 10^−10^/eV for light ions and of order 10^−11^/eV for heavy ions. A graphic representation of the typical magnitude of these shift terms is given in Figure 2. Here, the ion mass has been varied between *m* = 1 u and *m* = 240 u and typical Penning trap parameters have been chosen, *i.e.*, trapping voltages *U*_0_ between 10V and 1000V, magnetic fields *B*_0_ between 1 T and 10 T, and values of *d* = 1 cm, *B*_2_ = 10mT/mm^2^ and *C*_4_ = 0.5 have been assumed.

## 3. Energy-Dependent Oscillation Frequencies for Spectroscopy

### 3.1. Classical Picture

Out of the twelve dependences described by equation (4) and the matrices (8), (10) and (11), only dependences concerning stable motions can be used for the present purposes. Since the magnetron motion is an unstable drift, it is not well-suited for the concepts presented here. Apart from the discussion of the Larmor frequency this leaves four principle equations. They describe the relative frequency shifts as a function of oscillation energies for the axial and perturbed cyclotron motion.

(12) $$\Delta \omega_{+}/\omega_{+}(E_{z})=\left(\frac{-6C_{4}}{{\mathrm{qU}}_{0}}\;\frac{\eta^{2}}{2}-\frac{1}{2m\omega_{-}\omega_{+}}\;\frac{B_{2}}{B_{0}}-\frac{1}{2{\mathrm{mc}}^{2}}\right)\;E_{z}$$

(13) $$\Delta \omega_{+}/\omega_{+}(E_{+})=\left(\frac{6C_{4}}{{\mathrm{qU}}_{0}}\;\frac{\eta^{4}}{4}-\frac{1}{2{m\omega }_{-}\omega_{+}}\;\frac{B_{2}}{B_{0}}\eta^{2}-\frac{1}{{\mathrm{mc}}^{2}}\right)\;E_{+}$$

and

(14) $$\Delta \omega_{z}/\omega_{z}(E_{z})=\left(\frac{1}{4}\;\frac{6C_{4}}{{\mathrm{qU}}_{0}}-\frac{3}{8{\mathrm{mc}}^{2}}\right)\;E_{z}$$

(15) $$\Delta \omega_{z}/\omega_{z}(E_{+})=\left(\frac{-6C_{4}}{{\mathrm{qU}}_{0}}\;\frac{\eta^{2}}{2}+\frac{1}{2{m\omega }_{-}\omega_{+}}\;\frac{B_{2}}{B_{0}}-\frac{1}{2{\mathrm{mc}}^{2}}\right)\;E_{+},$$

where the first term describes the electric shift, the second term the magnetic shift, and the third term the relativistic effect, respectively. For the Larmor frequency we have the additional two dependences

(16) $$\Delta \omega_{L}/\omega_{L}\;(E_{z},\;E_{+})=\frac{1}{2{m\omega }_{-}\omega_{+}}\;\frac{B_{2}}{B_{0}}\;(E_{z}-\eta^{2}E_{+}).$$

All terms of order *η*^2^ or higher may be assumed too small in magnitude for a significant contribution to the coupling, since $\eta^{2}=\omega_{z}^{2}/\omega_{+}^{2}$ is typically of order 10^−2^ or smaller, see above.

Inserting relations (1) to (3) into Equation (4) and again using the hierarchy *ω*_−_ ≪ *ω_z_* ≪ *ω*_+_ ≈ *ω_c_*, the absolute frequency shifts in terms of trap parameters can be approximated by

(17) $$\Delta \omega_{+}\;(E_{z})=\left(-\frac{3}{{\mathrm{qB}}_{0}d^{2}}\;C_{2}C_{4}+\frac{d^{2}}{{\mathrm{mU}}_{0}}\;\frac{1}{C_{2}}\;B_{2}-\frac{1}{2{\mathrm{mc}}^{2}}\;\frac{{\mathrm{qB}}_{0}}{m}\right)\;E_{z}$$

(18) $$\Delta \omega_{+}\;(E_{+})=\left(\frac{3}{2}\;\frac{{\mathrm{mU}}_{0}}{q^{2}d^{4}B_{0}^{3}}\;C_{2}^{2}C_{4}-\frac{1}{{\mathrm{qB}}_{0}^{2}}\;B_{2}-\frac{1}{{\mathrm{mc}}^{2}}\;\frac{{\mathrm{qB}}_{0}}{m}\right)\;E_{+}$$

where again the first term describes the magnetic shift, the second term the electric shift, and the third term the relativistic effect, respectively. Similarly, for the axial frequency one finds

(19) $$\Delta \omega_{z}\;(E_{z})=\left(\frac{3}{2}\;\frac{1}{{\left({\mathrm{qU}}_{0}m\right)}^{1/2}d}\;C_{2}^{1/2}C_{4}-\frac{3}{8{\mathrm{mc}}^{2}}\;\frac{{\left({\mathrm{qU}}_{0}C_{2}\right)}^{1/2}}{m^{1/2}d}\right)\;E_{z}$$

(20) $$\Delta \omega_{z}\;(E_{+})=\left(-\frac{3U_{0}^{1/2}m^{1/2}}{q^{3/2}d^{3}\;B_{0}^{2}}\;C_{2}^{3/2}C_{4}+\frac{d}{{\left({\mathrm{mqU}}_{0}C_{2}\right)}^{1/2}\;B_{0}}\;B_{2}-\frac{1}{2{\mathrm{mc}}^{2}}\;\frac{{\left({\mathrm{qU}}_{0}C_{2}\right)}^{1/2}}{m^{1/2}d}\right)\;E_{+}.$$

And for the Larmor frequency this yields

(21) $$\Delta \omega_{L}/\omega_{L}\;(E_{z},\;E_{+})=\frac{1}{{\mathrm{qU}}_{0}C_{2}}\;\frac{B_{2}}{B_{0}}\;\left(E_{z}-\frac{U_{0}C_{2}m}{{\mathrm{qB}}_{0}^{2}}\;E_{+}\right).$$

For the remaining discussion, we will make use of a quantum description of the ion motion in the confining potential of the trap, since the focus will be on ions cooled to low energies. Especially for laser-cooled ions close to the quantum mechanical ground state, the quantized picture is necessary. For spectroscopic applications, we will restrict the discussion to effects in a magnetic bottle and therefore tune out the electric anharmonicity by corresponding choice of *U_C_*/*U*_0_ in Equation (6). A quantum description of effects due to electric imperfections is given in detail in [1].

### 3.2. Quantum Picture

We employ quantum mechanical first order perturbation theory to describe the energy shift of the confined ion due to the presence of the magnetic bottle by

(22) $$\Delta E(N_{+},\;N_{z},\;M_{S})\;=\langle N_{+}\;N_{z}\;M_{S}\left|H^{\prime }\right|N_{+}\;N_{z}\;M_{S}\rangle$$

where *H*′ is the perturbation Hamiltonian describing the influence of the magnetic bottle and *N*_+_ and *N_z_* are the quantum numbers of the modified cyclotron and the axial motion, respectively. *M_S_* is the spin quantum number corresponding to the particle spin *S⃗*. The oscillatory motion of the ion in the potential of the trap is quantized such that e.g. *N_z_* = 0 denotes the ground state axial motion of the ion corresponding to an energy of *E_z_* = *ħω_z_*(*N_z_* + 1/2). Details about this formalism can be found in [1]. The perturbation by the magnetic bottle is given by

(23) $$H^{\prime }={g\mu }_{B}\frac{\vec{S}}{\hbar }\;{\vec{B}}^{\prime }+\frac{q\vec{v}}{c}\;{\vec{A}}^{\prime }$$

where *A⃗*′ is the vector potential corresponding to the magnetic bottle field ${\vec{B}}^{\prime }=B_{2}\;((z_{0}^{2}-\rho_{0}^{2}/2){\hat{e}}_{z}-z{\vec{\rho }}_{0})$. *g* is the *g*-factor of the particle measuring its magnetic moment in units of the Bohr magneton *μ_B_* = *eħ*/(2*m_e_*). The vector potential is given by

(24) $${\vec{A}}^{\prime }=\frac{1}{2}\;B_{2}\left(z_{0}^{2}+\frac{\rho_{0}^{2}}{4}\right)\;{\hat{e}}_{z}\times {\vec{\rho }}_{0},$$

and yields a total perturbation energy of

(25) $$\begin{array}{lll} E(N_{+},\;N_{-},\;N_{z},\;M_{S}) & = & {g\mu }_{B}\;B_{2}\;\frac{\hbar }{m}\;\left(\frac{N_{z}+\frac{1}{2}}{\omega_{z}}-\frac{N_{+}+N_{-}+1}{\omega_{+}-\omega_{-}}\right)\;M_{S}\\ & + & \frac{q\hbar^{2}}{m^{2}c}\;B_{2}\;\frac{N_{z}+\frac{1}{2}}{\omega_{z}}\;\frac{1}{\omega_{+}-\omega_{-}}\;\left[\omega_{+}(N_{+}+\frac{1}{2})+\omega_{-}(N_{-}+\frac{1}{2})\right]\\ & + & \frac{q\hbar^{2}}{m^{2}c}\;B_{2}\;\frac{N_{+}+N_{-}+1}{2(\omega_{+}-\omega_{-})}\;\frac{1}{\omega_{+}-\omega_{-}}\;\left[\omega_{+}(N_{+}+\frac{1}{2})+\omega_{-}(N_{-}+\frac{1}{2})\right]\\ & - & \frac{q\hbar^{2}}{m^{2}c}\;B_{2}\;\frac{\omega_{+}+\omega_{-}}{{\left(\omega_{+}-\omega_{-}\right)}^{2}}\;(N_{+}+\frac{1}{2})(N_{-}+\frac{1}{2}). \end{array}$$

The shift of individual frequencies is then obtained by writing down the perturbation energy difference of adjacent energy levels in the corresponding degree of freedom, for example *ħω_z_*(*N*_+_, *N*_−_, *N_z_*, *M_S_*) = *E*(*N*_+_, *N_−_*, *N_z_* + 1, *M_S_*) − *E*(*N*_+_, *N_−_*, *N_z_*, *M_S_*). Using this and including the relativistic shift in terms of quantum numbers, the corresponding frequency shifts can, similar to Equations (17)–(20), be written as

(26) $$\Delta \omega_{+}\;(N_{z})=\left[\frac{\hbar }{m}\;\frac{B_{2}}{B_{0}}\;\frac{\omega_{+}}{\omega_{z}}-\frac{\hbar \omega_{+}}{2{\mathrm{mc}}^{2}}\omega_{z}\right]\;(N_{z}+\frac{1}{2})$$

(27) $$\Delta \omega_{+}\;(N_{+})=\frac{\hbar }{m}\;\frac{B_{2}}{B_{0}}\;\left[\omega_{+}(N_{+}+3)+\omega_{-}(N_{+}+\frac{1}{2})\right]-\frac{\hbar \omega_{+}}{2{\mathrm{mc}}^{2}}\omega_{+}(N_{+}+\frac{1}{2})$$

and

(28) $$\Delta \omega_{z}\;(N_{z})=-\frac{3\hbar \omega_{z}}{8{\mathrm{mc}}^{2}}\omega_{z}(N_{z}+\frac{1}{2})$$

(29) $$\Delta \omega_{z}\;(N_{+})=\left[\frac{\hbar }{m}\frac{\;B_{2}}{B_{0}}\;\frac{\omega_{+}}{\omega_{z}}-\frac{\hbar \omega_{z}}{2{\mathrm{mc}}^{2}}\omega_{+}\right]\;(N_{z}+\frac{1}{2}),$$

again using the frequency hierarchy *ω*_−_ ≪ *ω_z_* ≪ *ω*_+_ ≈ *ω_c_*. Employing Equations (1)–(3) similarly as before, we find the approximate frequency dependences expressed in terms of trap parameters and the quantum numbers by

(30) $$\Delta \omega_{+}(N_{z})=\left[\frac{\hbar }{m}\;\frac{B_{2}}{B_{0}}\;\frac{q^{1/2}\;B_{0}d}{{\left({\mathrm{mU}}_{0}C_{2}\right)}^{1/2}}-\frac{\hbar }{2{\mathrm{mc}}^{2}}\;{\left(\frac{q}{m}\right)}^{3/2}\;\frac{B_{0}}{d}{\left(U_{0}C_{2}\right)}^{1/2}\right]\;(N_{z}+\frac{1}{2})$$

(31) $$\Delta \omega_{+}(N_{+})=\frac{\hbar }{m}\;\frac{B_{2}}{B_{0}}\;\frac{{\mathrm{qU}}_{0}}{m}(N_{+}+3)-\frac{\hbar }{2{\mathrm{mc}}^{2}}{\left(\frac{{\mathrm{qB}}_{0}}{m}\right)}^{2}\;(N_{+}+\frac{1}{2})$$

and

(32) $$\Delta \omega_{z}\;(N_{z})=-\frac{3\hbar }{8{\mathrm{mc}}^{2}}\frac{{\mathrm{qU}}_{0}C_{2}}{md^{2}}\;(N_{z}+\frac{1}{2})$$

(33) $$\Delta \omega_{z}\;(N_{+})=\left[\frac{\hbar }{m}\;\frac{B_{2}}{B_{0}}\;\frac{q^{1/2}B_{0}d}{{\left({\mathrm{mU}}_{0}C_{2}\right)}^{1/2}}-\frac{\hbar }{2{\mathrm{mc}}^{2}}\;{\left(\frac{q}{m}\right)}^{3/2}\;\frac{B_{0}}{d}\;{\left(U_{0}C_{2}\right)}^{1/2}\right]\;(N_{+}+\frac{1}{2}).$$

For the dependence of the spin precession (Larmor) frequency *ω_L_* = *gμ_B_* *B*_0_*/ħ* on the axial and radial quantum numbers we find

(34) $$\Delta \omega_{L}(N_{z},\;N_{+})=\frac{{g\mu }_{B}B_{2}}{m}\;\left(\frac{N_{z}+\frac{1}{2}}{\omega_{z}}-\frac{N_{+}+1}{\omega_{+}-\omega_{-}}\right)\;\approx \frac{{g\mu }_{B}B_{2}}{m}\;\left(\frac{{\mathrm{dm}}^{1/2}}{{\left({\mathrm{qU}}_{0}C_{2}\right)}^{1/2}}(N_{z}+\frac{1}{2})-\frac{m}{{\mathrm{qB}}_{0}}\;(N_{+}+1)\right).$$

We will also use the dependence of the oscillation frequencies on the spin orientation of the trapped particle, since it is of relevance for example when the continuous Stern-Gerlach effect in a magnetic bottle is used to determine the magnetic moment of the bound electron, as will be discussed in section 4.2. Using the spin orientation energy *E_S_* = *g_J_μ_B_B*_0_*M_S_*, we find the dependence of the axial and modified cyclotron frequencies on the spin orientation by

(35) $$\Delta \omega_{z}\;(M_{s})=\frac{\hbar }{2m_{e}}\;\frac{\omega_{+}}{\omega_{z}}\;\frac{B_{2}}{B_{0}}\;\frac{e}{q}\;g_{J}\;M_{s}\approx \frac{\hbar }{2m_{e}}\;\frac{\mathrm{de}}{{\left({\mathrm{qmU}}_{0}C_{2}\right)}^{1/2}}g_{J}\;B_{2}\;M_{s}$$

(36) $$\Delta \omega_{+}(M_{s})=\frac{\hbar }{2m_{e}}\;\frac{\omega_{+}}{\omega_{+}-\omega_{-}}\;\frac{B_{2}}{B_{0}}\;\frac{e}{q}\;g_{J}\;M_{s}\approx \frac{\hbar }{2m_{e}}\;\frac{e}{{\mathrm{qB}}_{0}}\;g_{J}\;B_{2}\;M_{s}$$

where *m_e_* is the mass of the bound electron, *g_J_* is the *g*-factor corresponding to its magnetic moment and *M_s_* is the spin quantum number. These equations can similarly be used for free particles like electrons, protons (or antiprotons) by inserting the corresponding masses, charges and magnetic moments.

## 4. Application to Spectroscopy

Looking at equations (12) to (16), we find 10 energy-dependent frequency shift terms excluding the relativistic corrections. Out of those, four terms are of significant magnitude (*i.e.*, non-zero and not of order *η*^2^ or higher). They can be used for a detection of changes in the oscillation energy by observing the corresponding shifts in the oscillation frequencies. These terms are Δ*ω*_+_(*E_z_*, *B*_2_), Δ*ω_z_*(*E_z_*, *C*_4_), Δ*ω_z_*(*E*_+_, *B*_2_) **(group A)** and Δ*ω_L_*(*E_z_*, *B*_2_). Additionally, equations (35) and (36) give the two terms Δ*ω_z_*(*M_s_*, *B*_2_) and Δ*ω*_+_(*M_s_*, *B*_2_) **(group B)**. Finally, there are four relativistic correction terms of order 1 in *E*/*mc*^2^ in Equations (12)–(15) **(group C)**.

- Terms in group A can be used to detect changes in the oscillation energy of confined ions, e.g., due to laser cooling or heating, and thus serve as an electronic detector for optical photons.
- Terms in group B allow to determine a spin change of the system, e.g., of a single electron bound in an ion, and therefore can serve as an electronic detector for microwave photons which induce spin transitions. This is the basis also for the continuous Stern-Gerlach effect.
- The relativistic terms in group C make oscillation frequencies dependent on kinetic energies even for ideal confining fields, but are generally too small in magnitude for spectroscopic purposes. However, the “direct” relativistic mass effect due to *E* = *mc*^2^ allows to weigh internal excitation energies by the corresponding frequency shift, e.g., of nuclear isomeric states in ions.

Additionally, the dependence Δ*ω_L_*(*E_z_*, *E*_+_) can be used for a manipulation of the Larmor frequency, which may be of use in spectroscopy as in group B. The dependences in group A can alternatively be used to measure magnetic bottle strengths by electronic means: for known trap geometry and confining fields, the electric anharmonicity *C*_4_ can be chosen by variation of the voltage ratio *U_C_*/*U*_0_ such that it cancels the effect of the *B*_2_ term, see e.g., Equations (12) and (13). A scan of *U_C_*/*U*_0_ such that the total energy-dependence of the oscillation frequency vanishes, yields the corresponding *B*_2_.

Any of these possible applications relies on a detection of the corresponding oscillation frequency shift. For confined ions, the typical axial and radial frequencies are roughly of order MHz and can be measured electronically with high accuracy, as discussed in detail in [1,17,21,26]. Briefly, the oscillation of the trapped charged particles induces oscillating image charges in electrodes of the trap which produce a current through a connecting electronic (resonance) circuit. This signal even of a single ion can be amplified and Fourier-transformed to yield the ion oscillation frequencies. For cooled ions under suitable trapping conditions, relative frequency shifts of 10^−10^ can be detected by application of a phase-sensitive detection scheme as outlined in [25,26]. When the frequencies themselves are not measured, but only a shift is to be detected, one can thereby circumvent the Fourier limit and detect sub-Hertz changes in sub-second times [26].

### 4.1. Optical Spectroscopy Using Group A Terms (“Blind Spectroscopy”)

#### 

##### Transition Energies

**Figure 3.**
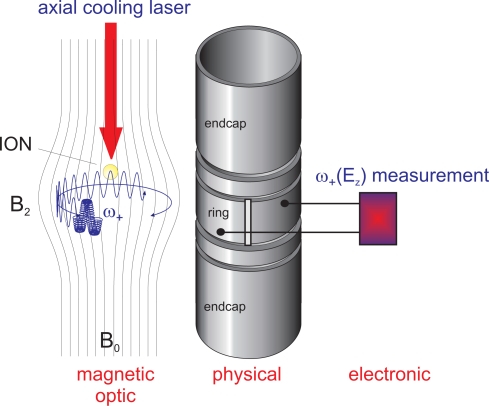
Illustration of the “blind spectroscopy” concept: a single stored ion is axially laser cooled on the optical transition of interest and the corresponding radial frequency shift is measured electronically. The cooling laser is scanned over the transition of interest and the resonance is detected as a maximum frequency shift.

Assume an ion stored in a magnetic bottle with *B*_2_ ≠ 0 superimposed to the magnetic trapping field *B*_0_. The terms Δ*ω*_+_(*E_z_*, *B*_2_) and Δ*ω_z_*(*E*_+_, *B*_2_) then describe the dependence of the radial frequency *ω*_+_ on the axial energy *E_z_* resp. the dependence of the axial frequency *ω_z_* on the radial energy *E*_+_ of the ion. The respective energies can individually be set to well-defined values by application of initial cooling, e.g., by resistive cooling to the cryogenic ambience temperature using a resonance circuit [17,21]. The effect of laser cooling (or heating) on an atomic transition of interest can then be observed by a frequency shift corresponding to the expressions Δ*ω*_+_(*E_z_*) or Δ*ω_z_*(*E*_+_). Scanning a narrow-band laser over the transition of interest, the resonance is found as a maximum shift of the corresponding ion oscillation frequency, which is detected electronically. The applicability and potential of such a scheme has been described in detail in [27]. Figure 3 schematically shows the measurement concept: in this example, a single stored ion is axially laser-cooled on the optical transitions of interest and the radial frequency shift Δ*ω*_+_ corresponding to the decrease of axial oscillation energy *E_z_* is measured electronically. Conceptually, in [27] the focus has been set on the precise determination of forbidden transition frequencies in highly charged ions (fine structure and hyperfine structure transitions), however, the concept is *per contructionem* applicable to any particle suited for laser cooling. The potential precision lies beyond the part per billion region due to the low ion velocities and details of the confinement [27]. The concept is applicable also to cases where ion production is difficult since only a single ion is needed. Also, it can be applied in transition frequency domains where suited photon detectors are unavailable, especially in the infrared.

##### Transition Rates

In a situation as described above, there are two mechanisms which can be used to change the energy of the ion oscillation in a well-defined way. One is the cooling or heating by the detuned laser, the other is the cooling or heating by a resonance circuit as used for initial resistive cooling. A balance between any two opposing mechanisms, e.g., laser heating against resistive cooling, results in a zero oscillation frequency shift as a function of time and may be used to determine the desired rate (inverse lifetime) Γ of the used optical transition. The power transferred to the ion by the laser is given by

(37) $$P_{\mathrm{opt}}=\hbar \Delta \omega \frac{S}{1+2S+{\left(\Delta \omega /\Gamma \right)}^{2}}\Gamma ,$$

where Δ*ω* is the laser detuning with respect to the atomic transition frequency and *S* = |Ω|^2^/Γ^2^ is the saturation parameter which is proportional to the square of the on-resonance Rabi frequency Ω. *S*/(1 + 2*S*) is equal to 1/2 for a fully saturated transition [2], and for sufficiently small laser detuning (Δ*ω*/Γ)^2^ ≪ 1, Equation (37) simplifies to

(38) $$P_{\mathrm{opt}}=\hbar \Delta \omega \Gamma /2.$$

For simplicity, we will use this relation for the further calculation. Depending on the sign of Δ*ω* this power transfer is positive or negative and can be balanced either by the (negative) power transfer of resistive cooling or the (positive) power transfer *P_E_* of electronic excitation via a resonant electric circuit, thus yielding the value of Γ by

(39) $$\Gamma =\frac{2P_{E}}{\hbar \Delta \omega }.$$

Another possibility, independent from electronic power transfer, makes use of the fact that the rate at which the observed frequency *ω*_+_ in the above example shifts is directly proportional to the desired transition rate Γ, since

(40) $$\frac{d}{\mathrm{dt}}\omega_{+}\propto \frac{d}{\mathrm{dt}}E_{z}=P_{\mathrm{opt}}\propto \Gamma .$$

Thus, for known detuning, saturation and magnetic bottle strength in a given trap, the desired transition rate can be determined by the rate of the frequency shift using

(41) $$\Gamma =\frac{1}{\hbar \Delta \omega }\frac{{\mathrm{mU}}_{0}C_{2}}{2B_{2}d^{2}}\frac{d}{\mathrm{dt}}\Delta \omega_{+}.$$

If these parameters are not known to sufficient accuracy, a system with well-known lifetime can be used to gauge the above relation and experimentally determine the proportionality factor between Γ and the frequency shift rate. The potential accuracy of this kind of lifetime measurement is limited by the accuracy to which the frequency shift rate can be measured, since all other parameters are well-controllable.

Alternatively, one can make use of the fact that the light pressure of laser cooling shifts the ion axially from the trap centre by an amount much larger than the motional amplitude. In the presence of a magnetic inhomogeneity, this results in a shift of the radial frequencies which can be measured. An axially asymmetric trapping potential may be used to restore the ion position and thus its radial frequencies, yielding the value of the shift. This directly determines the desired value of the transition rate Γ. The shift Δ*z* of the axial ion position is obtained from balancing the force *F_L_* of the laser with the restoring force *F_E_* due to the electrostatic trapping potential, *i.e.*.

(42) $$F_{L}=F_{E}\to \frac{\hbar \Delta \omega \Gamma }{2c}=\frac{{\mathrm{qC}}_{2}U_{0}\;\Delta z}{d^{2}}.$$

The result is

(43) $$\Delta z=\frac{\hbar \Delta \omega \Gamma d^{2}}{2{\mathrm{cqC}}_{2}U_{0}}.$$

This shift of the axial position (and thus the shift of the measured radial frequencies) can be restored if the electrostatic trapping potential is made asymmetric by introducing an additional voltage *U_A_* to one endcap. The effect of this is a shift of the axial position by

(44) $$\Delta z=\frac{1}{2}\;\frac{d}{z_{0}}\;\frac{U_{A}}{U_{0}}\;\frac{C_{1}}{C_{2}}\;d,$$

where *C*_1_ is the first term in the expansion of the trapping potential as given by equation (5) and is non-zero due to the introduced asymmetry. Equating the latter two expressions (43) and (44) yields

(45) $$\Gamma =\frac{{\mathrm{cqC}}_{1}}{\hbar \Delta \omega z_{0}}\;U_{A},$$

which is the desired transition rate expressed by the voltage *U_A_* applied to make the laser-induced shift of the axial position (and correspondingly of the radial frequency) vanish. Equation (45) has the nice feature that on the right hand side, we find only constants and well-controllable parameters. Also, Equation (45) is independent of the electric trapping potential given by *U*_0_ such that one is free to choose the axial frequency.

### 4.2. Microwave Spectroscopy Using Group B Terms

When an ion is confined in the presence of a magnetic bottle, the terms in group B (*i.e.*, Δ*ω_z_*(*M_s_*, *B*_2_) and Δ*ω*_+_(*M_s_*, *B*_2_)) provide that the ion oscillation frequencies depend on the spin orientation of an electron bound in the ion relative to the magnetic field. Especially for hydrogen-like ions, this so-called ‘continuous Stern-Gerlach effect’ offers a possibility to determine the spin orientation of the electron (which is an intrinsic ion property described by the magnetic spin quantum number *M_s_*) by a radiofrequency measurement of a macroscopic oscillatory motion of the ion. For typical confinement parameters, *i.e.*, for magnetic fields *B*_0_ of several Tesla strength, the Larmor frequency of electrons is in the microwave domain at typically 100 GHz, corresponding to photon energies of order meV. For protons, this number is still smaller by a factor of about 658, such that photon energies are of order *μ*eV and below. Irradiating such microwaves around the Larmor frequency of the spin precession around the magnetic field and scanning the microwave frequency across the Larmor resonance, the Larmor frequency can be found as a maximum spin transition rate [15].

Alternatively, the term Δ*ω_L_*(*E_z_*, *E*_+_) (equations (16) and (21)) may be used to scan the Larmor itself while keeping the irradiated microwave frequency fixed. The resulting resonance is equivalent to before, however this kind of ion manipulation is expected to be more difficult experimentally and limited in accuracy due to other energy-dependent effects coming into play. It may nevertheless be an option in selected cases, where the microwave frequency scan is impossible.

Using the terms in group B, the magnetic moment of the bound electron (and thus its *g*-factor *g_J_*) can be determined with a relative precision on the part per billion scale. Corresponding measurements have previously been performed on the hydrogen-like ions ^12^C^5+^ [9] and ^16^O^7+^ [10] and have provided stringent tests of bound-state quantum electrodynamics contributions to the theory value of the bound electron’s magnetic moment [15,28]. Also, they have provided the so far most precise value of the electron’s atomic mass [29]. Currently ongoing effort are directed towards similar measurements in medium-heavy and heavy, hydrogen- and lithium-like ions [30,31] as well as in free protons and antiprotons [30,32,33] with the goal of reaching even more stringent tests of theory contributions and possible determinations of fundamental constants [28].

### 4.3. “Weighing” Photons Using the Relativistic Mass Effect

The relativistic mass effect provides a change of the oscillation frequencies due to the mass change of the confined ion when its energy content is changed. The relativistic mass shift Δ*m* = Δ*E*/*c*^2^ changes the oscillation frequencies, such that the absorption or emission of a photon with energy Δ*E* can be monitored by an oscillation frequency measurement. Note, that it is not necessary to employ trapping field imperfections for a detection of relativistic effects such that this “weighing” of excitations is equivalent to high-precision mass measurements and has the same potential precision [34]. The relative frequency shift of order 10^−10^/eV for light ions is for optical spectroscopy at the limit of the current resolution, however, this does not restrict the principle idea. Absorption of a photon of several eV energy by a light ion may serve as a proof of principle. Given a sensitivity higher by an order of magnitude, the relativistic frequency shift could be a valuable tool in finding the famous low-lying nuclear transition in ^229^Th [35].

In highly charged ions, electronic excitation energies are much higher (up to order 100 keV) and could thus be detected much more easily, however the corresponding lifetimes of the excited states are extremely short. In few-electron ions, the upper state lifetime scales with the nuclear charge number *Z* as *Z*^−4^ for electric dipole, as *Z*^−6^ for magnetic dipole and as *Z*^−10^ for electric quadrupole transitions, such that only for *Z* < 5 lifetimes of order seconds are possible [36]. One well-known example is the metastable 2^3^S_1_ state in Li^+^ with a lifetime of about 50 seconds and a decay energy of about 60 eV [37].

In the study of nuclear de-excitations, however, both the upper state lifetime and the photon energies are potentially high. With photon energies in the keV to MeV region, the expected relative frequency shifts are of order 10^−7^ to several 10^−5^ and thus easily detectable. The corresponding ion recoil energy due to photon emission is given by *p*^2^/(2*m*), where *p* = *ħω*/*c* is the emitted photon momentum, and is of order eV for the highest relevant photon energies such that the confinement is not influenced significantly.

The radioactive decay of isotopes is followed by a discontinuous change of the mass-to-charge ratio of the ion (due to α or *β* emission) and can be detected as a corresponding frequency shift simply due to the relations (1) to (3).

**Figure 4.**
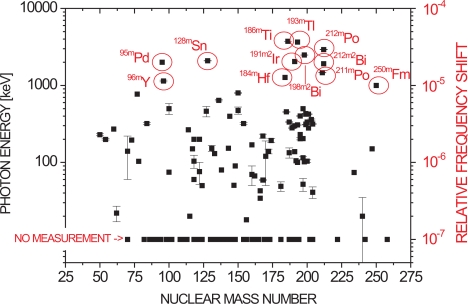
Metastable nuclear isomers with transition lifetimes between 1 and 1,000 seconds, for which either no reliable energy measurement exists and / or the transition energy is not known to better than 1 keV. Data taken from [38]. The encircled ions are particularly good candidates for trap-assisted measurements due to their large frequency shift of up to several 10^−5^.

The radiative decay of long-lived nuclear isomers, however, does not change the mass-to-charge ratio and can therefore only be seen by the relativistic mass shift corresponding to the emitted photon energy. Corresponding measurements of long-lived nuclear isomers have been performed, e.g., on ^65*m*^Fe [39] and ^68*m*^Cu [40]. Also, the *Q_EC_* value of the superallowed *β*-emitter ^26^Si has been determined by such a trap-assisted measurement with a relative accuracy of about 10^−5^ [41]. Suitable systems for trap-assisted measurements need to have isomeric lifetimes which are longer than the required frequency measurement time and are limited by the charge exchange lifetime *τ* of the ion due to residual gas in the trap, since a charge exchange alters the mass-to-charge ratio and makes a determination of the relativistic shift difficult or impossible. This upper limit is obtained by use of the semi-empirical Schlachter formula [42] for the charge exchange cross section and reads

(46) $$\tau =\frac{1}{3p}\;\frac{{\mathrm{mm}}_{g}}{m+m_{g}}\;\frac{q^{1.17}}{xI^{2.76}},$$

where *m* is the ion mass, *m_g_* is the mass of the residual gas particles, *I* is their ionization potential given in eV, *q* is the ion’s charge given in elementary units *e*, *p* is the residual gas pressure and *x* = 1.43 · 10^−16^m^2^. At typical cryogenic vacua, this lifetime is of order 1,000 seconds for highly charged ions. Correspondingly, Figure 4 shows all known metastable nuclear isomers with transition lifetimes between 1 and 1,000 seconds, for which either no reliable energy measurement exists and / or the transition energy is not known to better than 1 keV. This includes also the isomers for which no transition energy uncertainty is given. Together, these are about 170 out of 350 known isomers in the given lifetime interval. The data have been taken from [38]. The left hand scale gives the transition energy in keV, the right hand scale shows the corresponding relative frequency shift due to the relativistic mass effect. The encircled ions are particularly good candidates for trap-assisted measurements due to their large frequency shift of up to several 10^−5^. Assuming a frequency resolution of some 10^−10^, the transition energies of these nuclei can be measured with a relative accuracy of about 10^−5^, which is substantially more precise than any of the measurements shown in Figure 4. Due to the system-unspecific nature of the measurement method which does not require certain energy level schemes or a detection of the emitted photon(s), it is suited for any de-excitation process within the given lifetime region.

## 5. Summary and Outlook

We have discussed concepts for the detection of microwave, optical and X-ray photon absorption or emission by charged particles confined in a Penning trap. A common feature is the electronic and non-destructive measurement of oscillation frequency shifts in the radiofrequency domain following photon absorption or emission. Using specific inhomogeneities of the trap’s confining fields, the oscillation frequencies in the trap depend on the energy of the particles which is changed in photon absorption or emission. Thus, the particle oscillation serves as a sensor for photons and can be employed for spectroscopy. As examples, we have discussed the continuous Stern-Gerlach effect in the microwave domain, ’blind’ spectroscopy in the optical domain and the radiative de-excitation of nuclear isomers in the X-ray domain. These examples span over 12 orders of magnitude in the photon energy, from *μ*eV to MeV. Since the particle motion is confined and cooled in a well-defined way, and due to the high resolution of frequency shift measurements, the obtainable spectroscopic precision is high. It is of the order of 10^−10^ for the determination of magnetic moments using the Stern-Gerlach effect, potentially even beyond 10^−10^ for the determination of electronic transition energies using “blind” spectroscopy, and up to about 10^−5^ for X-ray spectroscopy of radiative de-excitation of nuclear isomers. The applications require transitions which can either be excited inside the trap or which are long-lived. On the other hand, only a single particle is needed and hence also rare species can be examined. The omission of direct photon detection makes the applications system-unspecific and reduces the experimental effort to already established electronic detection methods. The discussed applications serve for precision measurements of magnetic moments (*g*-factors), the energies and lifetimes of allowed and forbidden electronic transitions, and of nuclear transition energies also of highly charged ions, where bound-state quantum electrodynamics contributes significantly to spectroscopic properties. Such precision measurements therefore serve as a benchmark for QED theory, and, in turn, allow the determination of fundamental quantities like the fine-structure constant or the electron mass [28].
